# A survey of cancer affiliate network hubs in the US: goals, composition, resources, and evaluation

**DOI:** 10.1007/s10552-025-02070-8

**Published:** 2025-09-23

**Authors:** Madison M. Wahlen, Mary C. Schroeder, Pamela Y. Bojorquez, Sarah A. Birken, Jason T. Semprini, Jessica S. Gorzelitz, Aaron T. Seaman, Leila Sadri, Kristy Broman, Ingrid M. Lizarraga, Mary E. Charlton

**Affiliations:** 1 Department of Epidemiology, University of Iowa College of Public Health, Iowa City, USA; 2 Division of Health Services Research, University of Iowa College of Pharmacy, 180 South Grand Avenue, 346 CPB, Iowa City, IA 52242 USA; 3 College of Arts and Sciences, Loyola University Chicago, Chicago, USA; 4 Department of Implementation Science, Wake Forest University School of Medicine, Winston-Salem, USA; 5 Department of Public Health, Des Moines University College of Health Sciences, West Des Moines, USA; 6 Department of Health and Human Physiology, University of Iowa College of Liberal Arts and Sciences, Iowa City, USA; 7 Department of Internal Medicine, University of Iowa Carver College of Medicine, Iowa City, USA; 8 O’Neal Comprehensive Cancer Center, University of Alabama at Birmingham, Birmingham, USA; 9 Department of Surgery, University of Alabama at Birmingham, Birmingham, USA; 10 Department of Veterans Affairs, Birmingham VA Medical Center, Birmingham, USA; 11 Department of Surgery, University of Iowa Hospitals and Clinics, Iowa City, USA

**Keywords:** Cancer networks, Cancer care quality, Surveys and questionnaires

## Abstract

**Purpose:**

Community hospitals play a critical role in cancer care delivery across the US but face challenges in providing needed services and specialized expertise. Cancer affiliate networks, i.e., formal/informal relationships between a large cancer center (hub) and community cancer centers (affiliates), can potentially improve quality in small hospitals where many Americans receive care. However, published descriptions of these networks are lacking. Thus, we aimed to identify and describe cancer networks across the US.

**Methods:**

We assembled a listing of US-based cancer affiliate networks using a formal web searching strategy and surveyed hub representatives regarding network goals, composition, services/resources, and methods for evaluating success.

**Results:**

Sixteen of 21 identified networks responded to the survey. All networks focused on improving access/delivery of high-quality cancer care at affiliate sites with common goals that included improving access to services, supporting quality improvement, and recruitment for clinical trials. Networks often included multiple healthcare systems; few had financial ownership over affiliates; and most had participation requirements (e.g., accreditation). Hubs extended a variety of specialized services/resources to affiliates (e.g., clinical trials, genetic counseling, virtual tumor boards). Approaches to evaluation and quality improvement varied.

**Conclusion:**

We found that cancer affiliate networks generally aimed to improve access and delivery of high-quality cancer care, with considerable variation in the activities used to achieve and evaluate progress toward these goals. As networks continue to operate and emerge, research is needed to identify best practices for optimizing networks’ influence on the quality of care in resource-constrained hospitals and enabling effective scaling of the cancer affiliate network model.

**Supplementary Information:**

The online version contains supplementary material available at 10.1007/s10552-025-02070-8.

## Introduction

Non-academic, community hospitals serving their local catchment areas play a crucial role in cancer care delivery, with up to 85% of people diagnosed with cancer in the United States (US) relying on community hospitals for some aspect of their cancer care [1]. Community hospitals are often the preferred cancer care setting for patients, especially rural patients, who face financial and travel barriers to accessing care at larger, urban cancer centers [2]. Unfortunately, community hospitals, again particularly in rural areas, also face barriers to providing comprehensive cancer care due to resource constraints, staffing shortages, and limited access to specialty and supportive care providers [3–5]. Barriers to rural patients’ access are further amplified as cancer care in the United States becomes increasingly complex and multidisciplinary. For instance, more than 30% of rural residents live more than 180 min from a National Cancer Institute (NCI)-designated cancer center or an affiliated satellite location, demonstrating limited geographic access to large, well-resourced, specialized, and research-oriented comprehensive cancer services among rural patients [6]. Rural hospitals are less likely than urban hospitals to provide comprehensive oncology services including genetic counseling, nutritional support, or psychosocial counseling, and they often lack the infrastructure to support data monitoring, quality improvement initiatives, and multidisciplinary tumor boards [7–9]. Together, these challenges to accessing high-quality comprehensive cancer care may contribute to decreased receipt of guideline-concordant cancer care among rural patients and partly explain the worse clinical and patient-level outcomes observed in rural patients compared to urban patients [10–12].

One potential strategy to improve both access and quality of cancer care for rural patients is through a “cancer affiliate network” model, wherein the resources and specialized expertise available within large well-resourced cancer centers (“hubs”), typically located in metropolitan areas, are extended to community hospitals, often located in nonmetropolitan areas, through formal or informal partnerships. In the US, metropolitan areas are defined by the Department of Agriculture’s Economic Research Service as large labor-market regions with populations greater than 50,000 people, while nonmetropolitan areas refer to regions with smaller populations and can be further classified as rural and non-rural based on population size and adjacency to metropolitan areas [13]. This cancer affiliate network model has been successfully implemented in several regions across the globe [14, 15]. For instance, the Australian Comprehensive Cancer Network connects cancer centers across Australia including in rural and remote regions to Comprehensive Care Centers through a virtual integrated network in order to improve access to specialty services and clinical expertise for all cancer patients in Australia no matter where they are located [14]. Another example of a similar network model is the Princess Margaret Cancer Care Network in Canada which facilitates connections between partner institutions and the Princess Margaret Cancer Center, the largest Canadian comprehensive cancer center, to share best practices, improve patient access to clinical trials, and expand access to specialized clinical expertise and services [16]. One example in the US is the University of Kentucky Markey Cancer Center Affiliate Network (MCCAN) [15–18]. MCCAN was implemented to support the delivery of high-quality cancer care in community hospitals, many of which are rural or under-resourced. MCCAN leverages the resources of the University of Kentucky’s NCI-designated Markey Cancer Center to support cancer care (e.g., direct access to services, clinical trials recruitment, etc.) and assist affiliate community hospitals in achieving and maintaining American College of Surgeons Commission on Cancer (CoC) accreditation [15–18]. MCCAN affiliation is perceived as beneficial for improving cancer care quality by affiliate administrators and clinicians [15, 18] and is associated with improvement in multiple cancer treatment quality metrics [17]. MCCAN’s success suggests that the cancer affiliate network model is an effective intervention to address barriers to the delivery of high-quality cancer care in under-resourced areas.

Effective dissemination and scaling of the cancer affiliate network model requires an understanding of how cancer center networks operate in the US to identify, disseminate, and implement best practices. However, assessments and cataloging of cancer affiliate networks in the US and their characteristics are lacking. Though health system networks have been studied extensively [19–24], evidence specifically on cancer affiliate networks has been limited to studies evaluating the effect of network affiliate branding on clinical outcomes and patient decision-making [25–27]. There has been little attention to the networks themselves, including their organizational structure, goals, and operational processes. In this study, we sought to identify existing cancer affiliate networks across the US and describe the goals, composition, resources, and evaluation of these networks. Our findings represent the first evidence of which we are aware of the current state of US cancer affiliate networks, thus informing future studies to optimize networks’ influence on cancer care quality.

## Materials and methods

### Network identification and recruitment

Lacking published evidence of cancer affiliate networks, we leveraged our knowledge of MCCAN and similar networks to define a cancer affiliate network as a formal or informal relationship between a large cancer center and community cancer centers to achieve common goals [15, 28]. Using this definition, we identified cancer affiliate networks through three web searching strategies. As there is no published registry of cancer affiliate networks in the US, we used the following search terms and phrases in Google to identify them: “Comprehensive cancer networks,” “cancer networks AND affiliate programs,” “collaborative cancer networks,” “cancer networks AND affiliated partnerships,” “cancer network alliances,” and “cancer networks in the United States.” Next, we used the information provided by the NCI on their website [29] to identify NCI-designated cancer centers which might serve as potential network hubs. We scanned the information on the NCI website and individual program websites for the phrasing of “network(s),” “affiliate programs,” “community hospitals,” or other potential indicators of their involvement in a cancer network. Finally, we identified all members of the National Comprehensive Cancer Network (NCCN) [30] and searched each institution in Google, followed by the term “network” (e.g., “Duke Cancer Institute Network”) to identify additional networks. Networks were not required to include an NCI-designated cancer center or be a member of the NCCN to be considered for inclusion in the study. While multiple networks were known to the research team, all but one were identified through the web search strategy. The remaining network was also included in the study.

We verified whether the network met our definition (a formal or informal partnership between a large cancer center and community hospital affiliates) by reviewing cancer affiliate networks’ websites. A second team member then reviewed the websites to verify the search results. When there was disagreement, inclusion of a network was determined based on full research team consensus. Using contact information collected from the network websites, the senior author, who established relationships with several networks, recruited representatives with apparent leadership roles at the network hubs via email. One follow-up email was sent to nonrespondents after one week. The study’s goals were stated in the recruitment email, and initial contacts were asked to identify alternative representatives from their network hub if there was someone better suited to answer questions about their network. After representatives agreed to participate in the study, we sent a link to a brief online survey. All but one network had a single representative complete the survey; one network had two representatives complete the survey.

### Survey instrument development, data collection, and analysis

The survey instrument was designed to collect information about network goals, composition (i.e., financial relationship with affiliates, requirements for membership, etc.), resources (i.e., services extended to affiliates), and evaluation of network success (Online Resource 1). Response formats included multiple choice and short-answer text response. We refined the instrument based on feedback from survey methodologists and implementation scientists. We pilot tested the instrument with representatives from two cancer networks familiar to the research team. No changes to the survey instrument were required based on pilot testing, and these pilot data were included in final analyses.

The survey instrument was deployed using Qualtrics (Provo, UT), and data were exported to Excel for descriptive analyses (e.g., means, standard deviations). Qualitative text responses were reviewed and summarized. For consistency, manual changes to multiple choice survey questions were made as was appropriate based on data reported in optional, free-text responses. For instance, if a multiple choice option was not selected by the respondent, but was described in free text, that multiple choice option was manually selected by the research team and reflected in the final analysis. In the case of the network with two survey respondents, any positive response (i.e., multiple choice option selected) took precedence over a negative response (i.e., a multiple choice option not selected).

We used multiple publicly available sources to collect data to further characterize network hubs. NCI cancer center designation, NCCN membership, and CoC accreditation were confirmed by searching their respective websites [29–31]. Academic affiliation was defined as affiliation with a university or other academic institution and was gathered from primary institution websites. Metropolitan status was assessed using the address of the primary institution and defined using the US Department of Agriculture Rural Urban Continuum Codes [13]. This study was determined not to be human subjects research by our Institutional Review Board.

## Results

### Description of networks and respondents

Our search identified 21 networks meeting our definition, and we were able to contact representatives from 18. We sent surveys to all 18 networks, and representatives of 16 network hubs completed the survey. Individual respondents included network executives (i.e., program and medical directors) and administrators.

Network characteristics are described in Table 1. Almost all (94%) of the hubs were NCI-designated cancer centers with CoC accreditation and had an affiliation with an academic institution. Many (63%) were also NCCN member institutions. All hubs were in metropolitan counties. The number of affiliate sites in each network ranged from 2 to 22 sites (mean: 9). The networks surveyed collectively served 31 states across the US as reported by respondents. Some networks had affiliate sites in multiple states.

**Table 1:** Characteristics of networks and network hubs

	*N*	%
Networks	16	
Number of affiliate sites (mean, (range))^a^	9 (2–22)	
Number of states served (mean, (range))	2 (1–7)	
Network hubs	16	
NCI-designated cancer center	15	94
NCCN member institution	10	63
CoC-accredited cancer program	15	94
Academic affiliation	15	94
Metropolitan	16	100

*NCI* National Cancer Institute, *NCCN* National Comprehensive Cancer Network, *CoC* Commission on Cancer

^a^Data missing for one network

### Main network goals

Main goals varied across networks (Table 2), with all but one reporting multiple goals. The three most frequently cited goals included (1) improving access to cancer treatment, prevention, specialized services, etc. (*n* = 13 networks), (2) supporting quality improvement in cancer-related care (*n* = 12 networks), and (3) recruitment for clinical trials (*n* = 12 networks). One network included “sharing oncology best practices” as a main goal in free text.

**Table 2:** Network primary goals and organizational structure

	*N*	%
Primary goals		
Facilitating timely referrals	9	56
Recruitment for clinical trials	13	81
Research collaboration	12	75
Community engagement	11	69
Education for providers	11	69
Improving access to cancer treatment, prevention, specialized services, etc.	13	81
Supporting quality improvement in cancer-related care	13	81
Organizational structure		
Multiple healthcare systems	15	94
Financial ownership over some/all affiliate sites	4	25
Membership fee	9	75^a^
Affiliate requirements to join or remain in network	10	83^a^

^a^Denominator is based on networks without financial ownership over some/all affiliate sites (*n* = 12)

### Network structure

All but one network spanned multiple healthcare systems. Only four of the networks’ hubs had financial ownership over some or all affiliates (Table 2). Of the 12 networks that did not own their affiliates, 75% charged a membership fee and 83% had formal requirements of affiliates. Network requirements for participation included accreditation by various cancer- and noncancer-specific accrediting bodies such as the CoC and the Joint Commission, formal contracts/assessment processes and regular renewal, commitment to a charitable mission, and availability of clinical research trials (data not shown, free-text entries).

### Network services and resources for affiliates

Most networks’ hubs (75%) reported providing affiliate sites with access to specialized clinical services (e.g., genetic counseling, virtual second opinions, etc.) (Table 3). All but one offered access to clinical trials. Services most frequently provided to affiliates included genetic counseling and testing (83%), virtual tumor boards (83%), survivorship support (75%), subspecialty clinical care (67%), and psychosocial support (58%) (Table 2). Less commonly offered services included nutritional support (25%), integrative medicine (25%), inpatient chemotherapy (8%), rehabilitation (8%), and diagnostic services (17%). Additional services identified from free-text entries included patient education, a 24/7 patient navigation center, and quality improvement support (data not shown).

**Table 3:** Network resources and services

	*N*	%
Hub provides affiliates access to specialized clinical services	12	75
Specialized services^a^		
Genetic testing/counseling	10	83
Virtual second opinions	7	58
Virtual tumor boards	10	83
Nutritional support	3	25
Psychosocial support	7	58
Integrative medicine services	3	25
Subspecialty clinical care	8	67
Inpatient chemotherapy	1	8
Bone marrow transplantation	6	50
Diagnostic services	2	17
Pathology services	5	42
Access to clinical trials	11	92
Rehabilitation services	1	8
Survivorship support	9	75
Opportunities for training or continuing education (± continuing education units)^b^		
For providers/staff	16	100
For administrators	11	69
For ancillary staff	13	81
Strategies for sharing educational opportunities and resources^b^		
Webinars or symposiums on specific cancer-related topics of interest	15	94
In-person seminars on specific cancer-related topics of interest	13	81
Web-based portal	7	44
Access to grand rounds presentations at the primary institution	11	69
Shadowing opportunities for various health professionals	12	75
Network-wide conferences or meetings on specific topics	10	63

^a^Denominator is based on networks who provide access to specialized clinical services (*n* = 12)

^b^Denominator is based on networks who provide opportunities for training or continuing education (*n* = 16)

All network hubs offered affiliate providers/staff some form of training or continuing education, with or without formal credits (Table 3). One network noted, “We offer a very robust educational program for our affiliates on pertinent topics related to cancer care. [sic] Recently finished a 3-part CME series on treatment of benign heme diseases.” Most networks also provided learning opportunities for administrators and ancillary staff, 73% and 80%, respectively (Table 3). Networks commonly shared educational and resources with affiliates through webinars (100%) and in-person seminars (87%) on cancer-related topics, shadowing opportunities (80%), and access to Grand Rounds from the hub cancer center (73%) (Table 3). All networks employed multiple strategies simultaneously.

Most network hubs (69%) engaged in quality improvement activities in cancer-related care with affiliate sites (Table 4), and about half (56%) specifically monitored cancer care quality at their affiliate sites. Common quality improvement activities included assistance with meeting accreditation requirements for third-party accrediting bodies (e.g., CoC, American Society of Clinical Oncology, Quality Oncology Practice Initiative (QOPI)), process improvement, care coordination improvement, treatment delivery standardization, and addressing barriers to care.

**Table 4:** Network cancer care quality improvement activities

	*N*	%
Engages in some assessment of network activities and success	11	69
Quality improvement at affiliate sites		
Network monitors quality in cancer care at affiliate sites	9	56
Hub engages in quality improvement activities with affiliate sites	11	69
Quality improvement activities with affiliate sites^a^		
Improvement of care coordination	7	64
Monitoring of patient safety events	5	45
Standardization of treatment delivery	6	55
Process improvement	9	82
Monitoring and improvement of treatment adherence	6	55
Creation of patient education materials	5	45
Screening for and optimization of treatment of pain and other symptoms, including treatment side-effects	4	36
Evaluation and optimization of the patient experience	2	18
Addressing barriers to care, such as issues with transportation or financial barriers	6	55
Assistance with meeting required standards for third-party accrediting bodies (e.g., CoC, ASCO QOPI)	9	82

*CoC* Commission on Cancer, *ASCO* American Society of Clinical Oncology, *QOPI* Quality Oncology Practice Initiative

^a^Denominator is based on networks who engage in quality improvement activities with affiliate sites (*n* = 11)

### Evaluation of network success and affiliate quality

Qualitative responses to “How do you evaluate the success of your network?” varied widely. Eleven networks described their assessment of network success. Themes identified from the free-text responses included evaluating the impact of the network on (a) the hub (e.g., referrals, research accrual, etc.), (b) affiliates (e.g., stakeholder engagement/satisfaction), or (c) quality of care at affiliate sites (e.g., quality audits, measuring outcomes, etc.). Individual responses varied from one network to another. Network impact on the hub was evaluated by some networks using number of referrals, clinical trials accrual, number of collaborative research projects, number of programs initiated into the network, and whether the network is “financially successful” for both the hub and affiliates (not further elaborated). Impact on the affiliates was evaluated by some networks using patient and staff satisfaction surveys, clinician engagement, number of outreach events hosted and their impact, number of individuals engaged in professional education offered through the network, and number of specialized referrals to services not offered in the community. Finally, quality of care was evaluated by some networks using regular quality reviews and audits with affiliates, discussions with stakeholders, project-specific metrics, monitoring of quality metrics at affiliate sites, and monitoring patient outcomes through a state-based centralized cancer registry.

## Discussion

This study is the first to identify US cancer affiliate networks and describe them in terms of four domains: their goals, composition, resources, and evaluation practices. The lack of a national database of existing cancer affiliate networks and inconsistency in how networks present themselves online emphasizes the challenges in studying the cancer affiliate network model as well as the importance of understanding the role of these networks in community cancer care. Goals and activities cited across all networks included improving access to quality cancer care and extending resources and specialized services from hubs to affiliates. Most networks maintained financial independence from their affiliates. Despite these similarities, networks varied widely in how they operated, provided services, and evaluated network success, and there was considerable heterogeneity across the four domains from one network to another.

All networks we surveyed reported focusing on improving cancer care access and/or quality for their affiliates either directly through the hub providing specialized services to affiliates or indirectly through activities such as provider education/training, clinical trial access, and support for quality improvement activities. These types of services are often difficult for smaller community hospitals to provide [7–9]. Consistent access to high-quality, uniform cancer treatment may mitigate disparities in cancer experiences and outcomes [12]. Thus, these network activities may improve outcomes at affiliate sites, especially in under-resourced settings like rural hospitals, through facilitating access to resources and adoption of evidence-based practices. However, this is difficult to evaluate within existing cancer affiliate network models because only about half of the network hubs reported monitoring quality of care at affiliate sites. Prior studies evaluating cancer treatment outcomes within brand-sharing network affiliations have shown variability in quality between large hospitals and their affiliates [25, 27]. Therefore, evaluating the effectiveness of network activities in achieving desired goals is also important, and examining existing practices may help to identify successful strategies for doing so.

We found that networks commonly focused on improving access and quality of cancer care; however, there was considerable variation among individual networks with respect to specific goals, composition, operation, and evaluation, suggesting that the cancer affiliate network model may not be a “one size fits all” approach. This variability may be due in part to different contexts in which each cancer affiliate network operates and the unique needs of the populations they serve. At this time, it is unknown which aspects of cancer affiliate networks, if any, are essential to network success, or which elements of network success are context dependent. Systematic evidence of networks’ goals, structures, activities, and evaluation could be applied to innovative analytic approaches such as configurational comparative methods to identify combinations of conditions that drive network success, in turn informing the diffusion of innovative models [32].

This study is exploratory in nature and has several important limitations. Although we used multiple strategies to identify networks, including web searching strategies and professional connections, we likely did not capture all operational cancer affiliate networks meeting our definition. We were limited to cancer affiliate networks that advertise online with the keywords and phrases of our search strategy. Furthermore, there may be other large health systems that function as a cancer affiliate network but do not label or present themselves as such or prominently advertise their network online and would have therefore not been identified for our study. Additionally, in most cases, we only collected survey responses from a single representative from each network. In the one case where multiple representatives were surveyed, responses did vary. This study also focused on network goals and operation from the perspective of the network hubs. We were not able to elicit the affiliate hospitals’ goals or motivations for making the investment to join these networks, nor to clarify how the hub’s assessment of success aligned with those of the affiliate site. Previous work has shown that identification and support of individual affiliate hospitals’ goals by network leadership was a primary driver of one network’s effectiveness [18], demonstrating the importance of considering the affiliate perspective to understand network operation and success. Our study may also be limited by social desirability bias; survey participants may have overstated features of their networks. However, missing and highly varied responses suggest that participants accurately represented their networks. Finally, the survey format did not allow us to capture responses in great detail. A mixed-methods study using these survey responses to inform semi-structured qualitative interviews with network representatives is warranted.

Despite these limitations, a study describing cancer affiliate networks across the United States as we have defined it has never been published, and our findings have important implications for research, policy, and practice. It is unclear whether these networks generally improve quality of care despite the present study demonstrating this as a key network motivation. While evidence from one network suggests that network affiliation may improve adherence to evidence-based quality metrics [17], future research should focus on identifying and investigating relevant outcomes for evaluating network effectiveness such as quality of care, patient outcomes, and cost.

Cancer affiliate networks represent a strategic investment of scarce healthcare resources. We argue this investment warrants greater attention so policymakers can understand how investing in a network impacts quality, cost, and access to cancer care. From a policy standpoint, our study demonstrated that several networks engage in or require quality accreditations like CoC, QOPI, and Joint Commission. These endorsing organizations should also be aware of cancer affiliate networks, perhaps even considering network affiliation during accreditation processes. Additionally, 15 of 16 networks in this study were affiliated with an NCI-designated cancer center, presenting cancer affiliate network operation as an opportunity to expand community outreach and engagement activities as emphasized by the NCI [33–36]. Finally, these findings lay the groundwork for studies identifying best practices and successful implementation strategies of cancer affiliate networks and offer a foundation for network leaders to reflect on existing models, recognize potential gaps in planning or execution, and strengthen the design and function of their affiliation networks. As we continue to understand and evaluate whether cancer affiliate networks contribute to quality improvement, networks can learn from each other, and eventually network interventions can be developed and tested across diverse contexts to increase access to high-quality care and ultimately improve cancer outcomes for patients across the US and abroad.

## Supplementary Information

Below is the link to the electronic supplementary material.

Supplementary file1 (DOCX 427 KB)

## Data Availability

The data generated in this study are available upon request from the corresponding author.
