# Supplementary material for: A survey of cancer affiliate network hubs in the US: goals, composition, resources, and evaluation
Source: Cancer Causes Control. 2025 Sep 23;36(12):1919–26. doi: 10.1007/s10552-025-02070-8 (PMC12630224; doi:10.1007/s10552-025-02070-8)
Supplement: Supplementary file 1 — Supplementary file1 (DOCX 427 KB) [file 10552_2025_2070_MOESM1_ESM.docx]

**Title:** A Survey of Cancer Affiliate Network Hubs in the US: Goals, Composition, Resources, and Evaluation

**Journal:** *Cancer Causes and Control*

**Author list**

Madison M. Wahlen,^1^ Mary C. Schroeder,^2^ Pamela Y. Bojorquez,^3^ Sarah A. Birken,^4^ Jason T. Semprini,^5^ Jessica S. Gorzelitz,^6^ Aaron T. Seaman,^7^ Leila Sadri,^8^ Kristy Broman,^9,10^ Ingrid M. Lizarraga,^11^ Mary E. Charlton^1^

Affiliations

^1^ University of Iowa College of Public Health, Department of Epidemiology

^2^ University of Iowa College of Pharmacy, Division of Health Services Research

^3^ Loyola University Chicago, College of Arts and Sciences

^4^ Wake Forest University School of Medicine, Department of Implementation Science

^5^ Des Moines University College of Health Sciences, Department of Public Health

^6^ University of Iowa College of Liberal Arts and Sciences, Department of Health and Human Physiology

^7^ University of Iowa Carver College of Medicine, Department of Internal Medicine

^8^ University of Alabama at Birmingham, O’Neal Comprehensive Cancer Center

^9^ University of Alabama at Birmingham, Department of Surgery

^10^ Birmingham VA Medical Center, Department of Veterans Affairs

^11^ University of Iowa Hospitals and Clinics, Department of Surgery

**Corresponding author**

Mary C. Schroeder, PhD

Division of Health Services Research

University of Iowa College of Pharmacy

180 South Grand Avenue, 346 CPB

Iowa City, IA 52242

Email: [mary-schroeder@uiowa.edu](mailto:mary-schroeder@uiowa.edu)

Phone: (319) 384-4516

**Online Resource 1:** Survey instrument sent to cancer affiliate network hub representatives.


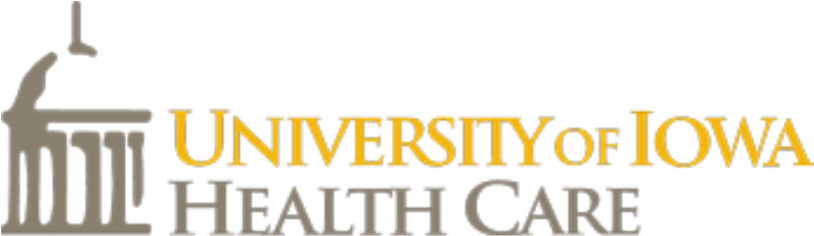


**Default Question Block**

This study aims to understand how cancer networks across the country operate and assist affiliate members in providing cancer care. The following questions will ask you about your cancer network's priorities, its composition, resources provided to affiliates by the network, and evaluation of the network’s success. This survey should take about 10 minutes to complete. Thank you for taking the time to help us learn more about your cancer network.

In the following questions, we will use the following terms:

- Cancer network: A formal or informal relationship between a large cancer center andcommunity cancer centers to achieve common goals.
- Primary institution: The cancer center which acts as the hub or resource center for thecancer network.
- Affiliate site(s): Community cancer centers (hospitals or clinics) with formal or informalmembership in the network.

What is your name?

What is the name of your cancer network?

What is your role within your cancer network?

Which of the following would you consider to be the main goals of your cancer network? **[Select all that apply]**

Facilitating timely referrals

Recruitment for clinical trials

Research collaboration

Community engagement

Education for providers (continuing education, national conference summaries/updates, shadowing

opportunities)

Improving access to cancer treatment, prevention, specialized services, etc.

Supporting quality improvement in cancer-related care

Other, please specify:

Does your primary institution have financial ownership over the affiliate sites?

Yes

No

Other financial relationship, describe:

I do not know

Is there a fee for affiliate membership (if sites are not owned by the primary institution)?

Yes

No

I do not know

Are there specific requirements of affiliate sites to join or maintain membership in the network?

Yes, please describe:

No

I do not know

Does your primary institution employ any of the clinical providers at the affiliate sites?

Yes

No

I do not know

Yes

No

Other (please describe):

I do not know

Does your network include affiliate sites from multiple health systems, or is it focused on a single health system?

Multiple health systems

Single health system

I do not know

Does the primary institution within the network provide access to specialized clinical services (e.g., genetic counseling, virtual second opinions, clinical trials, etc.) for patients of affiliate sites that are not otherwise available?

What specialized clinical services does the primary institution provide to affiliate sites? **[select all that apply]**

Genetic testing/counseling

Virtual second opinions

Virtual tumor boards

Nutritional support

Psychosocial support

Integrative medicine services

Subspecialty clinical care

Inpatient chemotherapy

Bone marrow transplantation

Diagnostic services

Pathology Services

Access to clinical trials

Rehabilitation services

Survivorship support

Other (please describe):

Does your network provide opportunities for training or continuing education to affiliates, either with or without continuing education credit? **[Select all that apply]**

Yes, for providers/staff

Yes, for administrators

Yes, for ancillary staff

Yes, other:

No

I do not know

What kind of educational opportunities do you offer? **[Select all that apply]**

Webinars or symposiums on specific cancer-related topics of interest

In-person seminars on specific cancer-related topics of interest

Portal access to network classes, workshops, CE programs, or web-based training or accreditation

resources

Access to Grand Rounds presentation at the primary institution

Shadowing opportunities for various health professionals

Network-wide meetings on specific topics

Other, please describe:

Do you offer any additional resources (aside from specialized services, research, or education) to your affiliates?

Yes, please describe:

No

I do not know

How do you evaluate the success of your network?

Does your network monitor quality in cancer care at your affiliate sites?

Yes

No

I do not know

How does your network monitor quality in cancer care at your affiliate sites?

Does your network engage in quality improvement activities in cancer-related care with your affiliate sites?

Yes

No

I do not know

What types of quality improvement activities in cancer-related care does the primary institution engage in with your affiliate sites? **[select all that apply]**

Number:

Improvement of care coordination

Monitoring of patient safety events

Standardization of treatment delivery

Process improvement

Monitoring and improvement of treatment adherence

Creation of patient education materials

Screening for and optimization of treatment of pain and other symptoms, including treatment side

effects

Evaluation and optimization of the patient experience

Addressing barriers to care, such as issues with transportation or financial barriers

Assistance with meeting required standards for third party accrediting bodies (e.g., Commission on

Cancer, ASCO QOPI)

Other, please describe:

How do you evaluate the success of quality improvement initiatives in cancer-related care at your affiliate sites?

**Last block**

How many affiliate sites make up your cancer network in this calendar year?

Which state(s) does your network serve? **[select all that apply]**

AlabamaMontana

AlaskaNebraska

ArizonaNevada

ArkansasNew Hampshire

CaliforniaNew Jersey

ColoradoNew Mexico

ConnecticutNew York

DelawareNorth Carolina

District of ColumbiaNorth Dakota

FloridaOhio

GeorgiaOklahoma

HawaiiOregon

IdahoPennsylvania

IllinoisPuerto Rico

IndianaRhode Island

IowaSouth Carolina

KansasSouth Dakota

KentuckyTennessee

LouisianaTexas

MaineUtah

MarylandVermont

MassachusettsVirginia

MichiganWashington

MinnesotaWest Virginia

MississippiWisconsin

MissouriWyoming

Powered by Qualtrics
